# Autoantibody Profiles in Autoimmune Rheumatic Diseases

**DOI:** 10.31138/mjr.30.2.86

**Published:** 2019-06-29

**Authors:** Nicola Bizzaro

**Affiliations:** Laboratorio di Patologia Clinica, Ospedale San Antonio, Tolmezzo, Azienda Sanitaria Universitaria Integrata di Udine, Italy

**Keywords:** Autoimmune rheumatic diseases, autoantibody profile, microarray, antinuclear antibodies, solid-phase assays

## Abstract

A paradigmatic feature of autoimmune rheumatic diseases (ARD) is the presence of multiple autoantibodies. The use of antibody profiles in the study of ARD therefore should be the best strategy for both diagnostic and classification purposes. To this end, systems using micronized components (protein chips or arrays), consisting of solid phase-linked autoantigens capable of simultaneously detecting many autoantibodies at the same time, are particularly suitable for testing autoantibody profiles. In the near future, extended disease-specific autoantibody profiles consisting of dozens, if not hundreds, of autoantibodies will be able to define each patient’s autoantibody fingerprint and identify subclasses of patients with different prognostic characteristics and different therapeutic responses.

## INTRODUCTION

The presence of circulating autoantibodies is a hallmark of autoimmune rheumatic diseases (ARD) and for more than 50 years their detection in biological fluids has represented a valid diagnostic aid for the rheumatologist. However, conventionally, the study of the autoimmune response has always been conducted by analyzing the presence or concentration of individual autoantibodies. The aim was to identify a few autoantibodies (often a single one), as specific markers of each autoimmune disease and to attribute to them a central role in the pathophysiology of each clinical picture. Today, the availability of analytical technologies able to measure more antibodies at the same time allows us to broaden our vision using autoantibody profiles, which is consistent with a paradigmatic feature of ARD: that of being diseases of multiple antibodies.

## THE EVOLVING SCENARIO OF AUTOIMMUNE DISEASE DIAGNOSTICS

**Figure 1.**
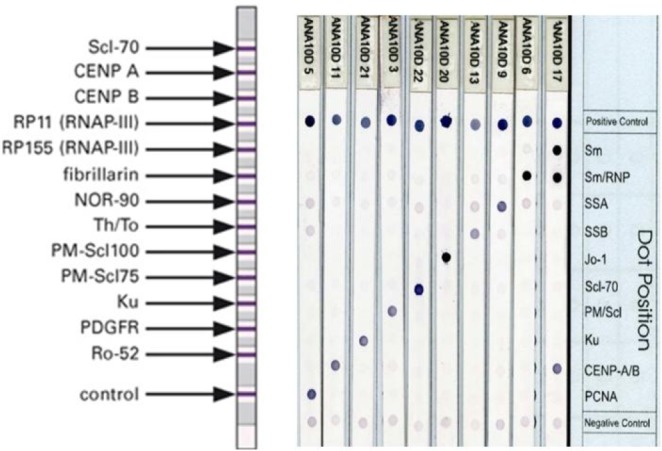
Commercial lineimmunoassay (left) and immunodotblot (right) methods to detect multiple antibodies (profiles) in autoimmune rheumatic diseases.

The modern autoimmunology laboratory is characterized by the presence of different analytical platforms that use manual methods or automated technologies. Alongside the well-established qualitative methods – such as indirect immunofluorescence (IIF) on HEp-2 cells for the detection of anti-nucleocytoplasmic antibodies (ANA) and on Crithidia luciliae for anti-dsDNA antibodies, and the quantitative immunometric methods (immunoenzymatic and immunochemiluminescent) used for the research of specific intracellular antibodies – new technologies have emerged such as immunoblot (IB) tests and line-immunoassays (LIA).^1^ Currently, IB and LIA are the most widely used methods to explore the antibody profile in ARD, enabling the simultaneous detection of 10–15 antibodies in one single step (*Figure 1*).

In recent years, by exploiting advances in proteomics, research has produced corresponding rapid advances in diagnostic technologies with the possibility of the simultaneous determination of hundreds of autoantibodies in the same reaction.^2,3^

Furthermore, in the wide and complex diagnostic field of ARDs, two aspects have to be considered that in recent years have profoundly changed the strategy of laboratory diagnostics. The first consists of the gradual consolidation of autoimmune diagnostics in a few laboratories with large volumes of activity, which has produced a necessary evolution towards fully automated technologies capable of processing many samples in a very short time.^4^ The second is due to the increased request for tests that were once almost exclusively requested by rheumatologists but that today are ordered by many other specialists and by family doctors. In these cases, tests are often ordered when there is a low pre-test probability – to rule out underlying ARD rather than to confirm an ARD. As a consequence, the positive predictive value of autoantibody test results has been greatly reduced, while its negative predictive value remains high. This is why the laboratory has to cope with these changes by providing screening profiles with high sensitivity to quickly discriminate negative results (about 70% of all antibody tests) and highly specific disease profiles, to confirm the results of the screening tests and to identify the antibody specificity.

## SCREENING PROFILES

**Figure 2.**
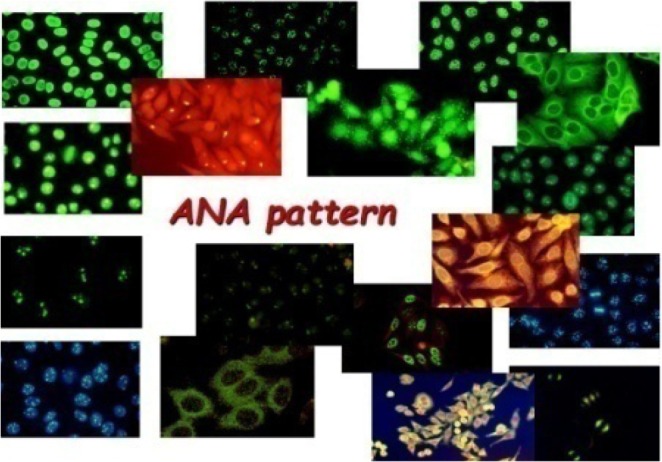
Most common antinuclear antibody patterns detected by indirect immunofluorescence on HEp-2 cells as a screening test for autoimmune rheumatic diseases and autoimmune liver diseases.

The term “screening profile” may seem like a contradiction of terms, since the autoantibody profile is usually sought at a later stage and as a result of a positive finding at the screening test.^5^ But, in truth, the most used screening test, the ANA test in IIF on HEp-2 cells, is already in itself a profile because it is made up of an array of cellular antigens able to detect a multitude of antibodies (*Figure 2*). However, interpretation of the ANA-IIF test is largely subjective and, as previously mentioned, the automation of the tests has become an unavoidable necessity. For this reason, in recent years, new fully automated solid-phase assays (SPA) capable of providing reliable and objective results in a short time have been introduced as an alternative to the ANA-IIF test.

Two solid-phase monotest assays, called CTD screen, are available today for ANA screening which include a mixture of 15–16 purified native or recombinant antigens among those most frequently recognized by autoantibodies in ARD. A recent review of all the studies comparing ANA-IIF to SPA has shown that screening ANA by SPA yields results that are at least comparable to—and probably better than—ANA-IIF results.^6^ These findings convincingly indicate that the CTD screen test can be advantageously combined with IIF when the ANA request has a low pre-test probability and when the requests do not report clinical information that would be useful for a targeted disease-oriented search for antibody specificity.^7^

### Autoantibody Profiling

The reasons that support the use of antibody profiles in the diagnostic framework of ARD are many. Antibody profiling can be advantageous for (early) diagnosis because it increases overall clinical sensitivity,^8^ since, in the very early stages of disease, the signs and symptoms do not always point to a single high pre-test probability of disease. Also, antibody profiling is advantageous for several other purposes: for classification, because it allows the definition of disease subtypes with different clinical manifestations; for prediction, because it may direct the diagnosis towards an ARD even in the early asymptomatic phases of the disease^9,10^ for prognostic evaluation, because the presence of certain antibodies is linked to the involvement of some organs and to the evolution of the disease; and, finally, for the purpose of determining a more personalized therapy, because the antibody profile could identify which subjects will be responsive or not responsive to a specific pharmacological treatment.^11^

In addition, there is a growing movement to classify patients based on a molecular taxonomy rather than on clinical grounds.^12^ For example, in a study of 260 subjects with idiopathic (autoimmune) inflammatory myositis investigated by LIA for 15 different myositis-specific autoantibodies, a multivariate cluster analysis showed that the single best determinant of disease cluster (dermatomyositis, anti-synthetase syndrome, inclusion body myositis and immune-mediated necrotizing myositis) was the presence of myositis-specific autoantibodies.^13^

In another study, a total of 3656 patients with systemic sclerosis classified into diffuse cutaneous (dcSSc) and limited cutaneous (lcSSc) subsets according to skin involvement were studied by the EULAR Scleroderma Trials And Research (EUSTAR) group.^14^ Upon multivariate analysis, several features were found to be independently associated with the prevalence of organ manifestations: scleroderma subsets, antibody status, and age at onset of Raynaud’s phenomenon. However, the clinical distinction was superseded by an antibody-based classification in predicting scleroderma complications.

Profiling the autoantibody repertoire using array-based technology has also emerged as a powerful tool for the identification of biomarkers in systemic lupus erythematosus (SLE). Autoantigen arrays carrying a wide variety of self-antigens – such as cell nuclear components (nucleic acids and associated proteins), cytoplasmic proteins, phospholipid proteins, cell matrix proteins, mucosal/secreted proteins, and other tissue-specific proteins – have been used to detect autoantibody specificities associated with particular manifestations of SLE.^15^

In a very recent study by Lewis et al.,^16^ a baculovirus-insect cell expression system was used to create an advanced protein microarray with 1543 full-length human proteins. Sera from 186 SLE individuals were assayed using the microarray: 68 novel proteins as autoantigens in SLE and 11 previously known autoantigens were identified. Using hierarchical clustering and principal component analysis, it was possible to classify four subgroups of SLE individuals associated with four corresponding clusters of functionally linked autoantigens: SLE 1a: original SLE autoantigens Ro60, La, and Sm complex; SLE1b: proteins involved in RNA, DNA and chromatin processing; SLE2: receptor-regulated SMAD (main signal transducers for receptors of the TGF-β superfamily); and SLE3: toll-like receptor pathways and lymphocyte development. A panel of 26 autoantibodies, derived by multinomial logistic regression taking into account the four identified SLE clusters, showed improved diagnostic accuracy compared to conventional antinuclear antibody and anti-dsDNA antibody assays. Of more relevant clinical significance, the authors found that autoantibody clusters were associated with different SLE manifestations: arthritis, thrombocytopenia, renal, pulmonary or neurological involvement.

Given the available evidence, it is very likely that further technological progress will substantially change the diagnostic approach to autoimmune diseases in the near future. Autoantibody profiles consisting of dozens if not hundreds of autoantibodies will be able to define each patient’s autoantibody fingerprint and identify subclasses of patients with different prognostic characteristics and different therapeutic responses.

## CONCLUSIONS AND FUTURE PERSPECTIVES

The development of new analytical technologies has made antibody profiling accessible to a greater number of laboratories, helping better define ARD. However, some questions remain unresolved because, although the aforementioned progress has helped to simplify the analytical phase, it has not resolved the problems related to the pre- and post-analytical phases. The challenges will be: (i) to limit the number of inappropriate test requests, both for economic savings, and (more importantly) to increase the predictive value of individual autoantibodies, according to Bayesian logic;^17–22^ (ii) to establish reflex procedures for diagnostic algorithms;^23^ and (iii) to actively collaborate in the interpretation and management of the results obtained, even in the presence of unexpected data.^24^ As underlined by Tozzoli,^22^ achieving these goals requires a constant commitment to training and information, the full knowledge of the various analytical technologies and autoantibody kinetics, the governance of all forms of automation and an attitude to multi-professional confrontation. In turn, great economic advantages in patient care and in the professional development of those working in autoimmune diagnostics can be expected.
